# Automatic Safety-Valve Stopper for Preventing the Bursting of Peroxide of Hydrogen Bottles

**Published:** 1901-07

**Authors:** 


### AUTOMATIC SAFETY-VALVE STOPPER FOR PREVENTING THE BURSTING OF PEROXIDE OF HYDROGEN BOTTLES.

THE great trouble with peroxide preparations (*National Druggist*, April,) is that if the containers are tightly corked, the oxygen which separates and is set free, accumulates slowly but constantly as time passes, until the bottles can no longer stand the pressure and burst, or the corks are driven out.

Containers of the hydrogen peroxide, U. S. P., which is a comparatively weak solution of $H_2O_2$, yielding but 10 volumes of oxygen, may be closed with a wooden stopper, which by the porous nature of the material, permits the escape of the gas almost as soon as it is set free, thus avoiding explosion and rupture of the bottles or the driving out of the corks.

While these wooden stoppers answer very well for solutions of $H_2O_2$ responding to 10 volumes of oxygen or less, with stronger solutions, such, for instance, as Marchand's peroxide of hydrogen medicinal (15 volumes), or his hydrozone (30 volumes of oxygen) they are quickly attacked by the solutions, as are also the ordinary corks, and within four months are completely oxidised, and rendered so soft that they cut like pot cheese.

In order to prevent these difficulties and especially to obviate the bursting of the bottles containing hydrozone, Mr. Marchand, the manufacturer of that article and other well-known brands of peroxide of hydrogen, has devised an ingenious stopper which he calls the automatic safety valve rubber cock.

The material of the stopper is vulcanised rubber. The beveled end is punctured through in such a manner that when the pressure in the bottle rises above 5 to 8 pounds to the square inch, the excess of free oxygen finds free egress and thus relieves the tension.

This device is first inserted, and a plug of porous wood is then driven in, thus stiffening the rubber and completing the operation of corking.

The capping consists of vegetable parchment covered with paraffined muslin, no wiring being used or needed, and there is no popping when corks are removed.

Retail druggists who have for so many years been the chief sufferers and losers from the bursting of the peroxide containers, and the deterioration of the substance otherwise from the causes indicated above, will welcome this device as a happy solution of what has to them been a very serious problem in the past, since it will enable them to supply their trade with the higher solutions of hydrogen peroxide.

The medical profession is being thoroughly advised of Mr. Marchand's new method of closing his bottles of peroxide of hydrogen medicinal and hydrozone, and will be certain to avail themselves of the advantages thus guaranteed them.

